# Dynamic causal model application on hierarchical human motor control estimation in visuomotor tasks

**DOI:** 10.3389/fneur.2023.1302847

**Published:** 2024-01-09

**Authors:** Ningjia Yang, Sayako Ueda, Álvaro Costa-García, Shotaro Okajima, Hiroki C. Tanabe, Jingsong Li, Shingo Shimoda

**Affiliations:** ^1^Research Center for Healthcare Data Science, Zhejiang Lab, Hangzhou, China; ^2^Department of Psychology, Japan Women's University, Tokyo, Japan; ^3^Human Augmentation Research Center, National Institute of Advanced Industrial Science and Technology, Chiba, Japan; ^4^Graduate School of Medicine, Nagoya University, Nagoya, Japan; ^5^Department of Cognitive and Psychological Sciences, Nagoya University, Nagoya, Japan

**Keywords:** dynamic causal model, functional magnetic resonance imaging, hierarchical motor control, visuomotor control, electromyography

## Abstract

**Introduction:**

In brain function research, each brain region has been investigated independently, and how different parts of the brain work together has been examined using the correlations among them. However, the dynamics of how different brain regions interact with each other during time-varying tasks, such as voluntary motion tasks, are still not well-understood.

**Methods:**

To address this knowledge gap, we conducted functional magnetic resonance imaging (fMRI) using target tracking tasks with and without feedback. We identified the motor cortex, cerebellum, and visual cortex by using a general linear model during the tracking tasks. We then employed a dynamic causal model (DCM) and parametric empirical Bayes to quantitatively elucidate the interactions among the left motor cortex (ML), right cerebellum (CBR) and left visual cortex (VL), and their roles as higher and lower controllers in the hierarchical model.

**Results:**

We found that the tracking task with visual feedback strongly affected the modulation of connection strength in ML → CBR and ML↔VL. Moreover, we found that the modulation of VL → ML, ML → ML, and ML → CBR by the tracking task with visual feedback could explain individual differences in tracking performance and muscle activity, and we validated these findings by leave-one-out cross-validation.

**Discussion:**

We demonstrated the effectiveness of our approach for understanding the mechanisms underlying human motor control. Our proposed method may have important implications for the development of new technologies in personalized interventions and technologies, as it sheds light on how different brain regions interact and work together during a motor task.

## 1 Introduction

Optimal feedback control (OFC) (1) in conjunction with brain theories such as the Bayesian brain hypothesis (2) and neural Darwinism (3) has been widely utilized to elucidate the mechanisms underlying action, perception, and learning. Beyond the traditional notion of functional localization (4), the prevailing concept for understanding brain functions involves the network interactions among various brain regions (5). Recognizing the significance of brain network connectivity is crucial for achieving optimal feedback control of movements, but the analysis of network dynamics during feedback motion control remains relatively unexplored. In particular, there has been little in-depth discussion of how network connectivity is altered during visuomotor feedback control when doing different voluntary levels of motion. This goes beyond conceptual ideas such as the dominant role of the cerebellum in feedforward control (6) and its contribution to muscle activity (7), or the strong activation of the motor cortex in voluntary movements (8).

Human motor control is achieved by minimizing the discrepancy between predicted and actual motor outcomes via sensory feedback (9). However, one fundamental challenge in understanding human motor control is elucidating how the motor cortex integrates feedback information and adjusts motor commands in order to reach an agent's targets (10, 11). Bayesian approaches address this challenge with hierarchical models that explain how signals pass through cortical sensory areas (12). The low-level controller is responsible for tuning the voluntary level during the entire motion process required for motor behavior, while the high-level controller is responsible for planning, instructing, and performing online correction of the low-level controller. Specifically, previous studies have shown that visuomotor adaptation tasks (13) are suitable for analyzing how the motor cortex integrates visual feedback information and interacts with the visual and cerebellar cortices. Based on that knowledge, we designed two voluntary levels of hand motion in target tracking tasks. The participant was asked to track the moving target with hand motions, which was different from the static target typically used in previous visuomotor tasks. We analyzed the network connectivity of several brain regions during the tasks with two different voluntary levels of motion to clarify the modulation of connectivity. The lower-voluntary-level motion was a more automatic hand motion involving up and down movement to track a target's movement without visual feedback of the motion, which represented motion dominated by the lower-level controller. The other was a higher-voluntary-level motion involving motor correction informed by moving targets and online visual feedback. This task elicited motion dominated by the higher-level controller. We used the online visual feedback to drive the participant to focus on the motor control task and evaluated the improvement of motor performance compared with the lower-level voluntary motion. We also verified the two levels of voluntary motion using muscle activation data (surface electromyography [EMG] in the upper extremity) to analyze the contribution of motor commands on muscle activity.

The development of non-invasive brain imaging techniques has enabled functional investigation of how neural systems achieve high- and low-level control in motor neuroscience. Dynamic causal modeling (DCM) is a suitable approach for studying brain interactions in motor control and verifying how they are affected by experimental conditions. DCM was proposed by Friston et al. (14) to explain effective connectivity among different brain regions and estimate hidden neuronal states based on measured brain activity, such as the blood oxygenation level-dependent (BOLD) signal using functional magnetic resonance imaging (fMRI). Model construction facilitates understanding of effective connections among different brain regions (15, 16) and adjusts for covariates (e.g., age, sex, body weight, and other behavior analysis results) to evaluate their potential effects on effective connections reflected in fMRI data (17). Several studies have used DCMs to analyze hierarchical motor systems (18), hierarchical planning (19), and cerebellar-premotor cortex interaction underlying visuomotor control (13). In this study, we implemented DCM to elucidate the connection between high- and low-level controllers and elucidate the modulation process. In addition to using DCM to analyze BOLD signals during the actions, we used surface EMG to identify the effects of different levels of motor control on muscle activation during different motion tasks. In detail, we employed this combined analysis to quantitatively evaluate individual differences in motor adjustment abilities, inspect information flows in hierarchical motor control systems, and ascertain how neural systems balance high and low-level control.

In this study, we aimed to understand the hierarchical human motor control in visuomotor adaptation using a combination of experimental behavior and DCM analysis. We first conducted online tracking tasks and fMRI group analysis to identify regions of interest in the motor, cerebellar and visual cortices that participated in the higher- and lower-level motor control. Then, we built a DCM to quantitatively reflect the intrinsic network and modulation among brain regions that contribute to fast-adaptation motor control. We hypothesized that motor-cerebellar interaction was affected by different levels of motor control that integrated visual information and modulated the effective connection strength. Furthermore, we tested whether effective connections could explain individual differences in the behavior analysis of tracking performance and the EMG analysis. We anticipate that effective modulation of connectivity on the motor-visual pathway by the higher-level motor task can contribute to effective task design in rehabilitation and training.

## 2 Experiments

### 2.1 Participants

Fourteen healthy right-handed participants (9 men, 5 women; mean age, 29.9±5.2 years; range, 24–44 years) took part in this study. Exclusion criteria included any history of motor injury or dysfunction, any MRI contraindication, pregnancy, history of brain injury, and claustrophobia. The experimental protocol was explained to the participants before the study. Both MRI experiments lasted about 2 h in total, and the EMG experiment lasted about 1.5 h, including setup, explanation, and testing. All participants provided written informed consent. This study was conducted in accordance with the Declaration of Helsinki and approved by the ethical review board of RIKEN (code of the ethical approval: RIKEN-W3-2021-023, date of approval: October 14th, 2021).

### 2.2 Experimental setup

**Figure 1:**
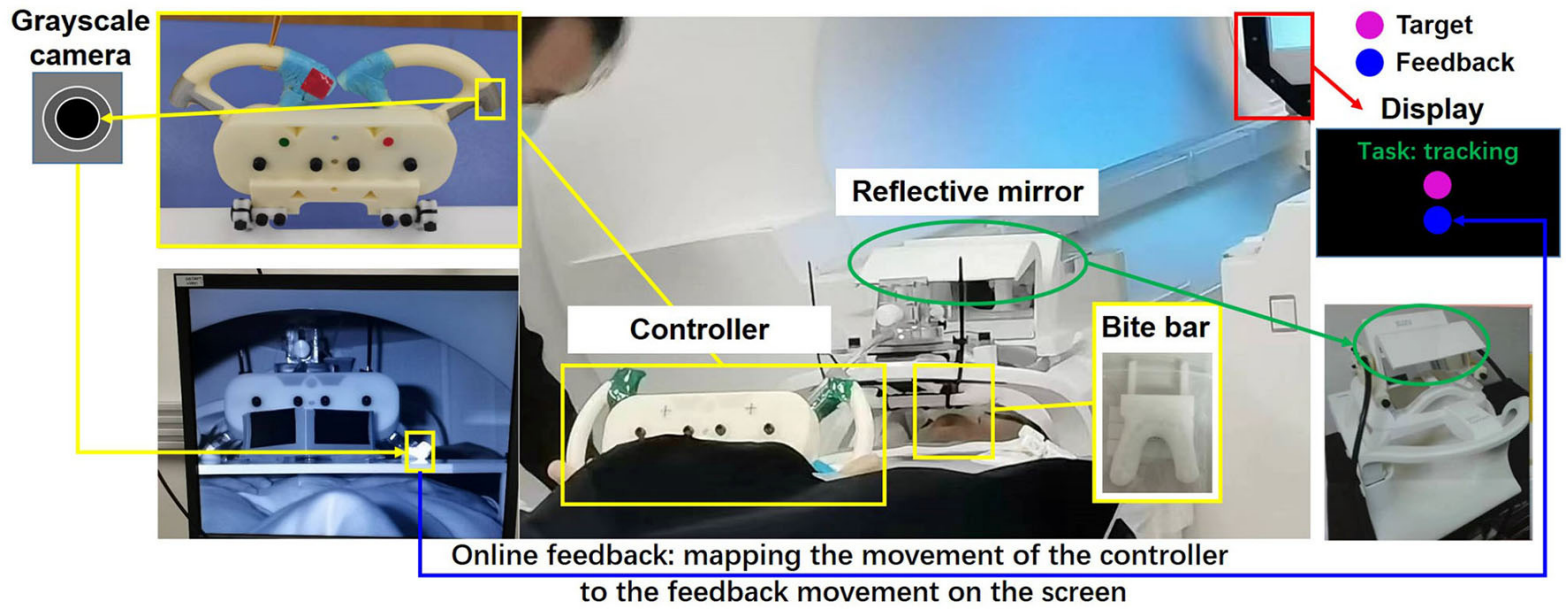
Experimental setting. Two closed-loop target-tracking tasks were designed and measured. The movement of the controller was represented by a passive reflective marker, recorded by a grayscale camera, and mapped online and shown on the screen to the participant.

The experimental motor tasks were two voluntary hand motion tasks: target tracking with no feedback (TTNF) and target tracking with feedback (TT). For each task, the participants were asked to use a 3D-printed small controller and track a target's reciprocating movement on the screen. The participants lay supine in the MRI machine and there was a small desk placed in front of their trunk, as shown in Figure 1. The 3D-printed controller was fixed on the table, and the table was adjusted to a suitable place for each participant. A bite bar was used to fix the position of the participant's head. Several sponge armrests were used to support the participant's arms. One display was placed above the participant's head and they could see the screen reflected in a mirror device on the head coil. Participants with myopia wore suitable glasses with corrective lenses provided by the MRI team. The display showed the experimental interface in a 1,280 × 720 pixels window with a black background. The target and feedback spheres in the TT task were shown in the middle of the window. The starting position for both spheres was 200 pixels from the bottom. The target sphere underwent vertical displacement on a screen with dimensions of 1,280 × 720 pixels, within a range of motion spanning from 200 to 600 pixels above the bottom of the screen. Each incremental motion step encompassed a distance of 25 pixels plus some inherent random variability falling within the range of [0–2] pixels, taken from a normal distribution. The task name and remaining rest time were displayed at the top of the screen. The 3D-printed controller was a simplified steering wheel and could only be moved up or down. In particular, the left and right parts of the controller were synchronized by a gear mechanism. On top of the right controller, there was a silver passively reflective marker that was used to represent hand movement. The movement of the marker was measured by a grayscale camera in the MRI room and mapped to the vertical movement of the feedback sphere within the same range of the target sphere. The task project was developed in Pycharm (Python 3.7.0) mainly using the OpenCV package. The movements of both feedback and target spheres were recorded, even in the TTNF task where the feedback sphere was not shown on the screen.

In the two motion tasks, the participants used the driving controller to perform reciprocating upper extremity motion. In the TTNF task, the participants could see only the target sphere on the screen. They used the controller and moved it up and down to follow the movement of the target sphere but received no feedback on their movements. Therefore, the TTNF task represents intentional motion without feedback, which was used to represent a lower-level motor control task. In the TT task, the participants could see both the target and feedback spheres. They needed to control the feedback sphere and track the movement of the target sphere. The TT task represented intentional motion with visual feedback. It required fast correction during the motion in order to obtain better tracking performance and thus represented a higher-level motor control task.

The measurements were carried in three sessions, and each session comprised three TTNF tasks and three TT tasks in random order. The duration of each task was 21 s and the screen showed the task name on the top middle part, above the sphere movement region. There was a 15 s resting time between each task. The screen also showed the text “Rest:” along with a countdown in 1 s increments.

### 2.3 EMG data acquisition

**Figure 2:**
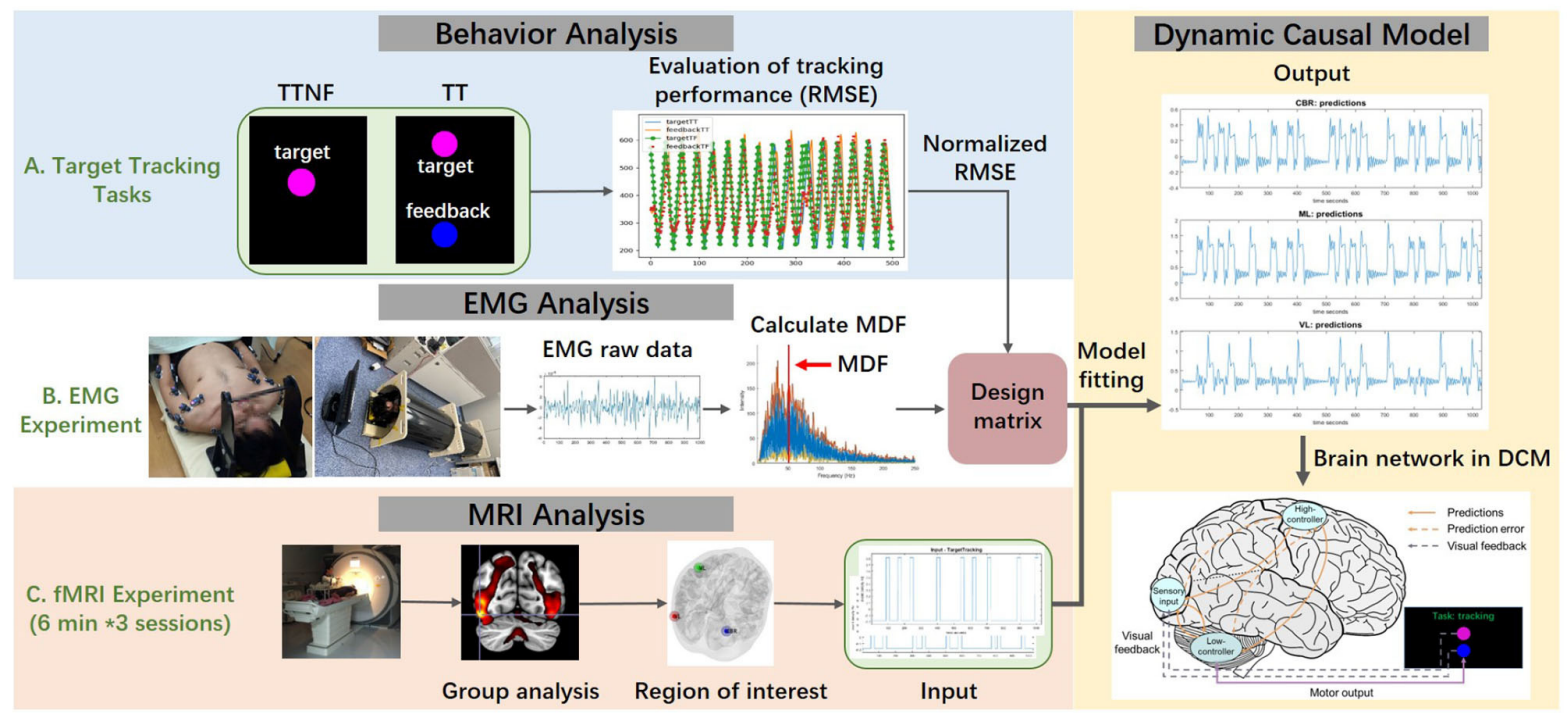
Scheme of the proposed method. **(A)** Behavior data were analyzed to evaluate the tracking performance during the TT task (target with feedback) and TTNF task (target with no feedback) by calculating the root mean square error (RMSE) between target and feedback spheres. **(B)** The electromyography (EMG) experiment used a replica of the functional magnetic resonance imaging (fMRI) experiment environment. Muscle activation in the upper extremity was recorded under the same experimental protocol. Raw EMG data were preprocessed to remove noise. Then the muscle median frequency and amplitude were calculated to assess individual differences. **(C)** fMRI measured the blood oxygenation level-dependent (BOLD) response during the motion tasks and clarified the region of interest using group-level analysis. Then we integrated the data and built the dynamic causal model (DCM) in order to understand how the high- and low-level controllers integrate the visual information and affect motor outputs.

We used the same experiment protocol for the EMG experiment in another measurement because EMG sensors could not be used in the MRI room. We created a replica of the MRI environment as shown in Figure 2B and measured the muscle activation of participants using the same posture to complete the hand motion tasks. To ensure the participants did similar motions as in the MRI measurement, we restricted their movements by fixing their body posture. In detail, we fixed the positions of the head, elbows, and wrists. To quantitatively evaluate the effects of brain activity on muscles, we measured surface EMG data from the right arm. A wireless surface EMG device (BTS Bioengineering Corp.) was used in this experiment to obtain the muscle activity data from the right side of the body at 2,000 Hz. Eight muscles related to upper extremity motions were measured based on their contributions to the extension and flexion of the wrist, elbow, and shoulder: (1) flexor carpi radialis (FCR); (2) extensor carpi radialis longus (ECRL); (3) brachioradialis (BR); (4) biceps muscle (BCM); (5) triceps muscle (TCM); (6) anterior deltoids (AD); (7) posterior deltoid (PD); and (8) superior fibers of the trapezius (SFT).

### 2.4 MRI data acquisition

As the first part of the proposed method, we obtained brain activities related to motion tasks using BOLD fMRI techniques. The brain fMRI images were acquired on a SIEMENS Prisma 3T system equipped with a 20-channel head and neck coil, a 32-channel spine coil, and a 4-channel small flex coil. The 84 slices in the brain had a field of view of 240 × 200 *mm*^2^ and a voxel size of 2.0 × 2.0 × 2.0 *mm*^3^ (echo time = 31 *ms*, repetition time = 1,910 *ms*, flip angle = 78 deg, and GRAPPA acceleration factor = 3, gap = 0 mm). Dummy scans were discarded. High-resolution (1 × 1 × 1 *mm*^3^, gap = 0 mm). T1-weighted anatomical images were acquired using a 3D-MPRAGE sequence (sagittal slice orientation, repetition time = 2.3 s, echo time = 2.7 ms, flip angle = 8 deg, FOV = 234 × 224 *mm*^2^, GRAPPA acceleration factor = 3).

## 3 Methods

### 3.1 Proposed method

To clarify the mechanism of human hierarchical control, we employed brain fMRI, muscle activity, and behavior analysis in voluntary hand motion during intentional tracking tasks with and without feedback. The proposed method is shown in Figure 2. We measured brain fMRI and computed the activation levels of the regions that had strong BOLD responses. We made a general linear model (GLM) of the two motor tasks along with the contrast, and we found that the visual cortex was not significantly activated during target tracking in the TTNF task. This suggests that the activity of the visual cortex is suppressed during motion, likely due to the limited information in this condition; detailed results are shown in Section 4.3. Conversely, the motor cortex and cerebellum showed activation in both conditions. These initial experiments led us to hypothesize that the network activity of the visual cortex, motor cortex, and cerebellum could be key to understanding the differences in these motions. Also, we were interested in the motor control of high- and low-voluntary-level motion and we observed that activation in the motor and cerebellar cortices was different. Based on these observations, we selected three volumes of interest (centered in the significant results from the GLM) in motor, cerebellar and visual cortices, which respectively serve as the higher-level controller, lower-level controller, and sensory layer in our hypothesized model. Then, we used DCM to investigate the dynamic interactions in our model.

We also measured EMG data from the upper extremity under the same experimental protocol in a simulated MRI environment. We calculated the median muscle frequency (MDF) to represent human muscle activation related to behavior control. These EMG data represented the effects of brain motor commands on muscle activation. Finally, we combined the behavior analysis results and fMRI and EMG analysis to understand how high- and low-level motor controllers integrate visual information and how they affect human behavioral performance.

### 3.2 Experimental behavior analysis

In this study, we recorded the positions of the target and feedback spheres during the two tasks at 30 Hz, although the feedback sphere was not shown to the participants on the screen during the TTNF tasks. To assess the tracking performance between the two conditions (with and without feedback), we calculated the root mean square error (RMSE) between the target position (*z*_*t*_) and feedback position (*y*_*t*_) of the two tasks, as in Equation 1. We hypothesized that the RMSE would be smaller in TT than TTNF because participants could fast-correct their tracking behavior and adjust the motor control command based on the visual feedback from the feedback sphere.


(1) $$\begin{array}{l} RMSE=\sqrt{\frac{1}{T}\overset{T}{\underset{t=1}{\sum }}{\left(z_{t}-y_{t}\right)}^{2}}, \end{array}$$

### 3.3 EMG preprocessing and analysis

We defined two types of voluntary intentional motion and evaluated the motion performance using behavior analysis. To further classify the motions, we applied muscle activation analysis to the EMG measurement data of the upper extremity. For preprocessing, all of the EMG signals were filtered with several notch filters (60 Hz, 120 Hz, 180 Hz, and 240 Hz) to remove power line interference and with a bandpass filter between 8 Hz and 200 Hz (20). After filtering, the data were divided into repetitions (one repetition represented a reciprocating movement). We used both amplitude (RMS) and MDF as features for assessing the effects of motor control on muscle activation. For the MDF calculation, we first calculated the MDF of individual muscles and then computed the mean MDF across the eight muscles measured. The mean MDF represented a global measure of muscle activity to assess the effects of the two target tracking motions on muscles, as given by Equation 2.


(2) $$\begin{array}{l} \overset{MDF}{\underset{j=1}{\sum }}P_{j}=\overset{M}{\underset{j=MDF}{\sum }}P_{j}=\frac{1}{2}\overset{M}{\underset{j=1}{\sum }}P_{j}, \end{array}$$

where *P*_*j*_ is the EMG power spectrum at frequency bin *j* and *M* is the width of the frequency bin. *M* is usually defined as the next power of 2 from the length of EMG data in the time-domain (21). We normalized the amplitude and MDF for each participant because of individual differences. We calculated the average of amplitude and MDF across the repetitions for each muscle for each participant in the TTNF condition. Here we show MDF normalization as an example, as in Equation 3. $\bar{MDF_{s}^{k}}$ represents the average of MDF across the repetitions for each muscle in participant *s*. *k* = 1, …, 8 indicates the index of the muscle. *t* = 1, …, *n* shows the repetition number, and *n* is the total number of repetitions. Then we used the average value of amplitude and MDF for normalization. Finally, we used the t-test to analyze whether the amplitude or MDF significantly differences between TTNF and TT.


(3) $$normMDF_{s}^{k}(t)=\;\frac{MDF_{s}^{k}(t)}{\bar{MDF_{s}^{k}}}=\frac{MDF_{s}^{k}(t)}{\sum_{t=1}^{n}MDF_{s}^{k}(t)/n}$$

### 3.4 MRI data preprocessing

SPM12 (22) (Wellcome Trust Centre for Neuroimaging, London, UK) was used to preprocess the brain fMRI and anatomical images and to perform statistical analysis. First, we applied slice time correction and re-alignment (rigid-body motion correction with six degrees of freedom) to the fMRI data. The phase encoding direction for the EPI sequence corresponded to the y-direction. It was defined as positive from the posterior to the anterior of the head, the same as the SPM default setting. We co-registered the mean EPI (fMRI) to T1 structural image, and then we applied segmentation and normalization of fMRI to Montreal Neurological Institute (MNI) template brain image, which wrapped data from each participant to MNI space. Next, the images were smoothed with an 8 mm full width at half-maximum isotropic Gaussian kernel. Finally, we performed model specification and group analysis [family-wise error (FWE) *p* < 0.05, cluster-level] to investigate the differences in BOLD brain responses among intentional motions with and without feedback and voluntary motion.

### 3.5 Dynamic causal model

To clarify the effective connectivity among motor and visual areas and the cerebellum, we employed DCM to modulate the neuronal activation among these brain regions. Each region is represented by one time-dependent output ż corresponding to the observed BOLD signals in this region. Here, we used a bilinear deterministic DCM that modulates the neuronal activation as in Equation 4. Matrix **A** represents the average effective connectivity from one region to another and matrix **B**^*i*^ specifies the modulation of effective connectivity due to experimental condition *i* = 1, 2, …, *m*. Each matrix **B**^*i*^ is multiplied by experimental inputs *u*_*i*_ relating to the experimental condition *i*. In this experiment, we had two **B**-matrices corresponding to *m* = 2 experimental conditions: TTNF (only an automatically moving target sphere) and TT (both target and feedback spheres). We set TTNF as the base condition. Therefore, all the parameters in matrix **B**^1^ were set as 0. Matrix **C** is the sensitivity of each region to driving inputs. Matrix **x**∈ℝ^*j*^ represents the measured neuronal activity of selected region *j*. The extraction of *x* from fMRI data was described in the “Regions of interest (ROIs)” part below. Three regions were selected in this study based on the fMRI group analysis results. Based on our research setting, we simplified Equation 4 to a specific modeling, as shown in Equation 5.


(4) $$\dot{X}=(A+\Sigma_{i=1}^{m}u_{i}B^{i})x+\Sigma_{i=1}^{m}Cu_{i},$$

(5) $$\left[\begin{array}{c} \dot{x_{1}}\\ \dot{x_{2}}\\ \dot{x_{3}} \end{array}\right]=\left[\begin{array}{ccc} a_{11} & a_{12} & a_{13}\\ a_{21} & a_{22} & a_{23}\\ a_{31} & a_{32} & a_{33} \end{array}\right]\left[\begin{array}{c} x_{1}\\ x_{2}\\ x_{3} \end{array}\right]+u_{2}\left[\begin{array}{ccc} b_{11}^{2} & b_{12}^{2} & b_{13}^{2}\\ b_{21}^{2} & b_{22}^{2} & b_{23}^{2}\\ b_{31}^{2} & b_{32}^{2} & b_{33}^{2} \end{array}\right]\left[\begin{array}{c} x_{1}\\ x_{2}\\ x_{3} \end{array}\right]+\left[\begin{array}{cc} c_{11} & c_{12}\\ c_{21} & c_{22}\\ c_{31} & c_{32} \end{array}\right]\left[\begin{array}{c} u_{1}\\ u_{2} \end{array}\right],$$

#### 3.5.1 Regions of interest

**Figure 3:**
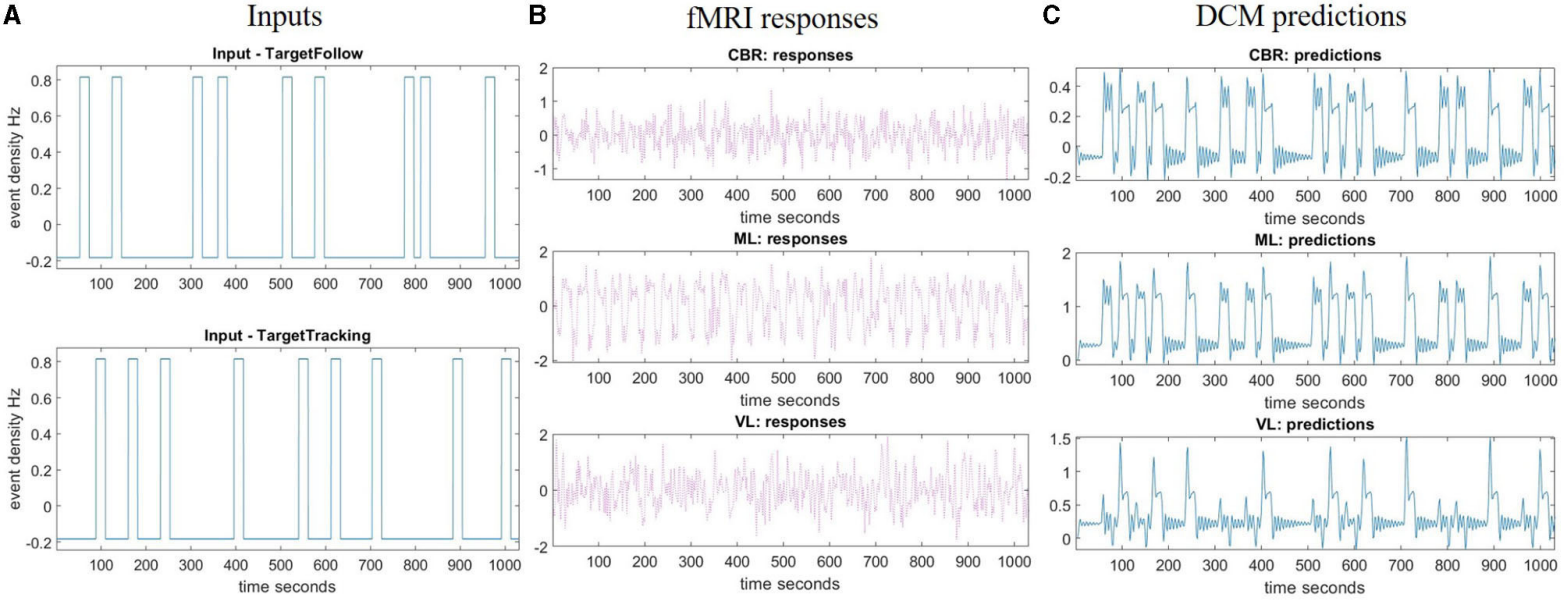
Region of interest analysis and DCM fitting example. **(A)** Inputs represent the task time for the TT task (target with feedback) and TTNF task (target with no feedback) during the measurement time. **(B)** Time series extraction. This is the first eigenvariate of the pre-whitened, high-pass filtered and confounded-corrected time series in the selected region (right cerebellum, left motor cortex, and left visual cortex). **(C)** Predictions made by DCM about the first eigenvariate values in the selected region. fMRI, functional magnetic resonance imaging; ML, left primary motor cortex; VL, left visual cortex; CBR, right cerebellum; DCM, dynamic causal model.

We restricted the DCM analysis to three brain regions: primary motor areas, visual areas, and the cerebellum. These regions were chosen to find the underlying connections between visual feedback and fast-corrected motion. To extract time series from significant voxels in each ROI (**x**∈ℝ^*j*^, as in Equation 4) in the TT > Rest contrast, subject-specific sphere centers were defined as the closest suprathreshold voxel (*p* < 0.001, uncorrected) to the MNI coordinates obtained from group-level analysis over all participants. For each subject, the three sessions of measurements were combined into one session. An “Effects of Interest” F-contrast was defined to tell SPM which regressors in the design matrix were of interest. Then, the individual time series were computed as the first eigenvariate across all suprathreshold voxels within 6 mm, as shown in Figure 3.

#### 3.5.2 DCM specification and Bayesian model selection

**Figure 4:**
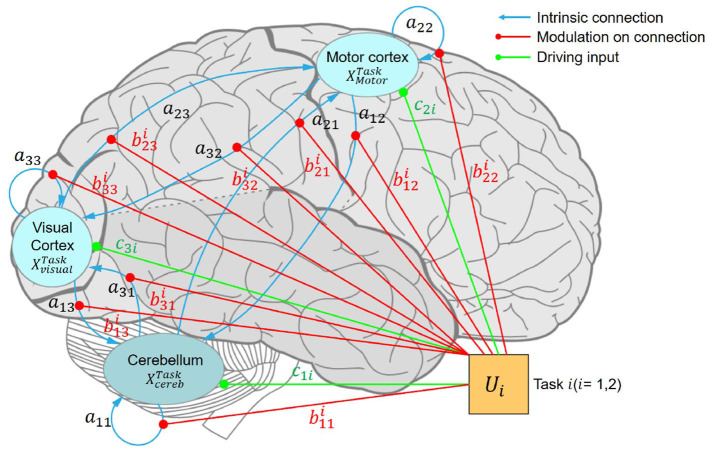
Fully connected DCM. Schematic illustration of a hierarchical motor control model comprising three regions of interest: the primary motor cortex, visual cortex, and cerebellum. ML, left primary motor cortex; VL, left visual cortex; CBR, right cerebellum.

First, we specified a fully connected DCM template as shown in Figure 4. The black lines represent the internal connections between different regions as in matrix *A*. The red lines in Figure 4 show the condition-related effects on the connections as in matrix *B*. The orange lines show the sensitivity of ROIs to driving inputs as in matrix *C*. This DCM template was fitted with time series extracted from ROIs among all the participants (23). Rather than declare any specific prior models, we searched among the reduced parametric empirical Bayes (PEB) models to find the best one by switching off the connections between different brain regions (17). This procedure compared the evidence for reduced models obtained by Bayesian model reduction by iteratively discarding parameters that do not contribute to the model evidence (24, 25).

Then, a Bayesian model average (BMA) is calculated over the models from the final iteration of the greedy search and the parameter estimates of the reduced model were obtained. In this paper, we showed the parameters with a posterior probability (PP).

#### 3.5.3 Design matrix

A group-level design matrix was used to describe the differences between participants as covariates (e.g., differences of RMSE in behavior analysis). All covariates were mean-centered and z-scored to avoid the problem of multicollinearity (17). The design matrix was modeled by a GLM with the group-level parameter estimates to evaluate the potential effects of these differences between participants. Finally, we performed a leave-one-out cross-validation (LOOCV, SPM toolbox, function spm_dcm_loo.m) to evaluate the predictive validity of the DCM.

## 4 Results

### 4.1 Visual feedback affects tracking performance

**Figure 5:**
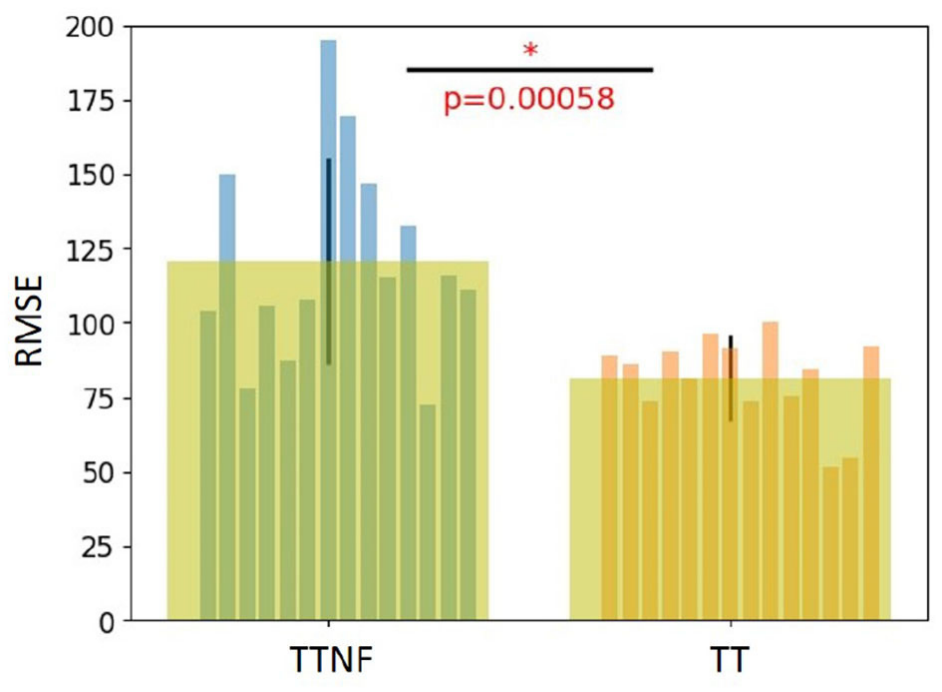
Behavior analysis results. The root mean square error (RMSE) of motor performance was calculated for the TT task (target with feedback) and TTNF task (target with no feedback). A significant difference in RMSE between them indicates that visual feedback improved the tracking performance in the behavioral experiment. The symbol “*” indicates a statistically significant difference in the comparison between the two groups (*p* < 0.00058).

We used RMSE to evaluate the tracking results of the TT task (target with feedback) and TTNF task (target with no feedback). The results are shown in Figure 5. For the TT task, we computed the RMSE between the target position and feedback position to evaluate the tracking performance of each participant. For the TTNF task, although the feedback was not shown on the screen, we still recorded the position of the feedback. We used the recorded feedback position and target position to evaluate the participants' tracking performance during the non-feedback motion task. The RMSE value was significantly smaller in the TT task (81.5±14.4) than in the TTNF task (120.8±34.7) (*t*-test, *n* = 14, *p* < 0.05). This indicates that participants could fast-correct their tracking behavior according to the visual feedback and thus decrease the RMSE with better tracking performance. The improved RMSE values from TTNF to TT were normalized and then used as covariates to adjust for individual differences in the DCM. We found a positive correlation (–0.52, *p* = 0.07) between the normalized improved RMSE and the estimated value based on the ML → ML connectivity in the DCM (for details, see **Table 4** and subsection “Behavior analysis and DCM evaluation” in the Discussion). This result suggests that increased activation in the motor cortex was related to improved RMSE, which indicated that precise motor control led to better tracking performance.

### 4.2 Muscle activation responding to different motion tasks

We used the *t*-test to examine whether the TTNF and TT tasks affected muscle activation differently. Table 1 shows the MDF results normalized by the mean MDF of each participant during the TTNF task in the relevant muscles. We found that four of the eight muscles measured (FCR, BR, TCM, and SFT) had significantly higher MDF in the TT task compared with the TTNF task (*t*-test, *p* < 0.05, uncorrected). In addition, the mean MDF across all eight muscles also showed significantly higher MDF in TT (*t*-test, *p* < 0.05, uncorrected). By contrast, PD showed significantly lower MDF in TT (0.991±0.097) compared with TTNF (1.000±0.100). No significant difference was found in the other three muscles measured (ECRL, BCM, ADS). The mean MDF was used as a covariate in group-level DCM fitting to evaluate the effects of individual differences. Table 2 shows the statistical analysis results for EMG amplitude normalized by the mean for the relevant muscles of each participant during TTNF. We found that, with the exception of muscle SFT, all seven other muscles had significantly increased amplitude during TT compared with TTNF (*t*-test, *p* < 0.05, uncorrected).

**Table 1:** Muscle median frequency.

**Muscle**	**1. FCR**	**2. ECRL**	**3. BR**	**4. BCM**	**5. TCM**	**6. ADS**	**7. PD**	**8. SFT**	**Mean**
*p* < 0.05	*		*		*		*	*	*
TTNF ave.	1.000	1.000	1.000	1.000	1.000	1.000	1.000	1.000	1.000
TTNF std.	0.229	0.099	0.103	0.065	0.058	0.054	0.100	0.092	0.041
TT ave.	1.059	1.007	1.032	1.004	1.009	0.996	0.991	1.010	1.014
TT std.	0.200	0.103	0.136	0.068	0.082	0.074	0.097	0.093	0.044

The symbol “^*^” denotes a statistically significant difference in the comparison between the two tasks, TTNF and TT, based on a *t*-test with a significance level of *p* < 0.05 (uncorrected).

**Table 2:** Muscle amplitude.

**Muscle**	**1. FCR**	**2. ECRL**	**3. BR**	**4. BCM**	**5. TCM**	**6. ADS**	**7. PD**	**8. SFT**	**Mean**
*p* < 0.05	*	*	*	*	*	*	*		*
TTNF ave.	1.000	1.000	1.000	1.000	1.000	1.000	1.000	1.000	1.000
TTNF std.	0.459	0.291	0.191	0.189	0.096	0.154	0.145	0.145	0.110
TT ave.	1.117	1.137	1.098	1.113	1.029	1.024	1.017	1.011	1.068
TT std.	0.654	0.683	0.258	0.236	0.155	0.313	0.175	0.123	0.187

The symbol “^*^” denotes a statistically significant difference in the comparison between the two tasks, TTNF and TT, based on a *t*-test with a significance level of *p* < 0.05 (uncorrected).

### 4.3 Brain activation in response to different motion tasks

**Figure 6:**
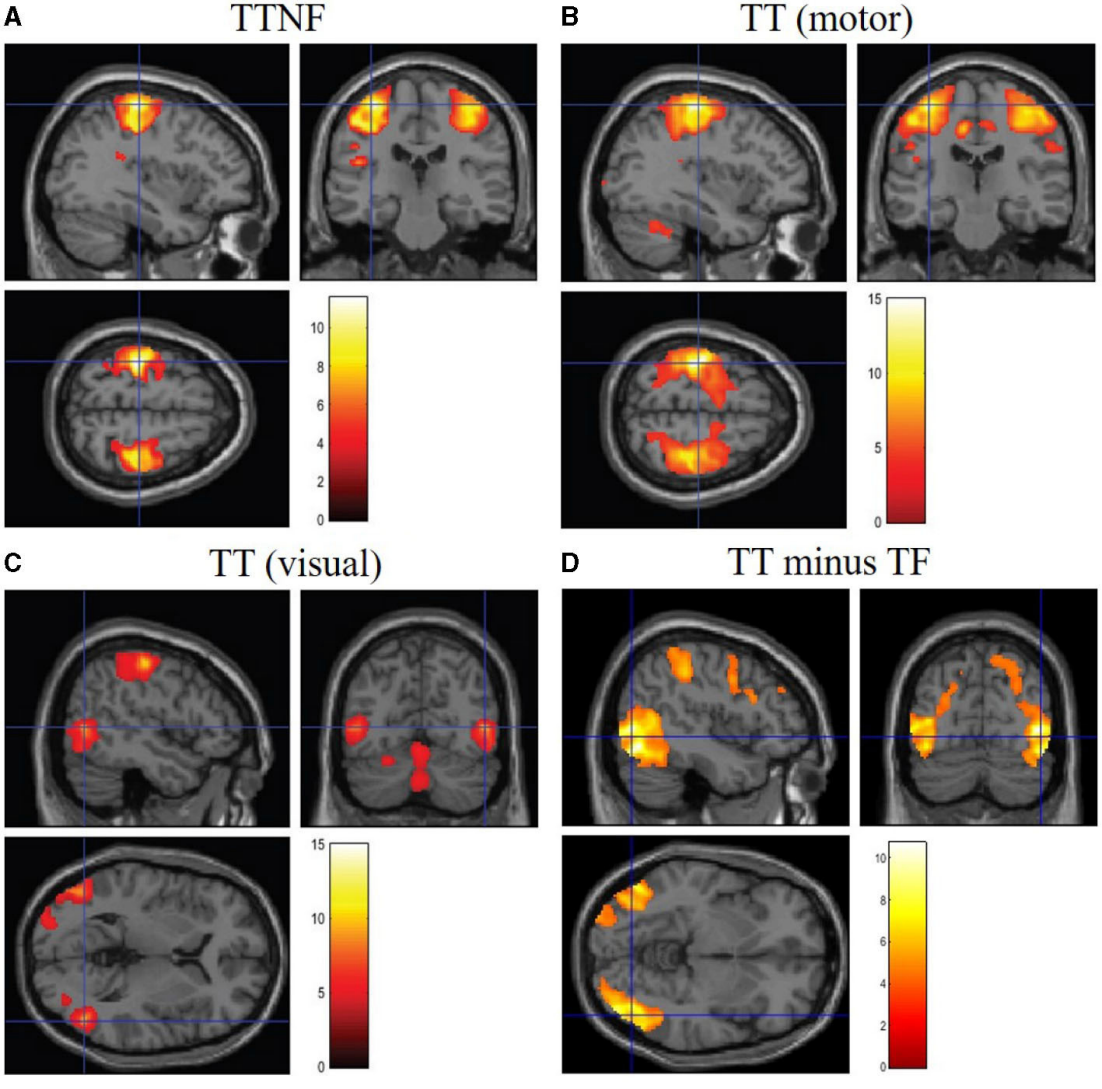
BOLD responses in the brain. Significance level setting: group analysis (*n* = 14), *p* < 0.05, family-wise error-corrected. **(A)** Significantly activated regions in the motor cortex during the TTNF task (target with no feedback). **(B)** Significantly activated regions in the motor cortex during the TT task (target with feedback). **(C)** Significantly activated regions in the visual cortex during TT task. **(D)** Contrast between TT and TTNF to highlight the differences.

To investigate the brain regions related to higher- and lower-level motor control movements, we analyzed the BOLD responses in fMRI data. We first analyzed the BOLD responses that were significantly higher than those in the resting state during the different motion tasks (Figure 6, group analysis: FWE *p* < 0.05, cluster level). The results show that the left and right sides of the motor cortex were activated separately (left: 2,378; right: 1,649 voxels; Figure 6A) during the TTNF task, while a larger motor cortex region (13,395 voxels, Figure 6B) that combined both sides was activated in the TT task. Besides the differences in the motor cortex, the activated region in the cerebellum was also larger in TT compared with TTNF (TT: 1,211 voxels; TTNF: 831 voxels). Moreover, there was no significant activation in the visual cortex in TTNF compared with the resting state, but both sides of the visual cortex were highly activated in the TT task (left: 365 voxels; right: 592 voxels), as shown in Figure 6C. We also made a direct contrast between TT and TTNF to highlight the differences in the visual cortex, as shown in Figure 6D. For an overview, see Table 3. We restricted the DCM analysis to the dominant (right) side and selected three ROIs (*p* < 0.001, uncorrected, sphere centers are the same as the group analysis results for TT contrast shown in Table 3) to represent the neuronal activation. These were the motor cortex and visual cortex in the left hemisphere, and the cerebellum in the right hemisphere, considering that all participants are right-handed and the left and right sides of the controller are interconnected via a gear mechanism.

**Table 3:** Group analysis of fMRI results.

**Brain region**	**Left hemisphere**					**Right hemisphere**				
	**x**	**y**	**z**	*k*	**t-value**	**x**	**y**	**z**	*k*	**t-value**
Primary motor cortex (TTNF)	–36	–24	62	2,470	11.56	34	–16	70	2,258	11.06
Primary motor cortex (TT)	–36	–22	62	11,146	14.96	32	–16	70	11,146	11.37
Visual cortex (TT)	–52	–72	0	837	8.92	50	–66	4	869	9.53
Cerebellum (TTNF)	–10	–50	–20	2,183	11.31	20	–46	–26	2,183	8.01
Cerebellum (TT)	–26	–38	–34	2,648	10.31	20	–46	–24	2,648	6.25

Group analysis: family-wise error *p* < 0.05, cluster level. *k* is the cluster size. x, y, and z are shown in Montreal Neurological Institute coordinates [mm]. For the significantly activated regions in the cerebellum during the TT task (target with feedback) and TTNF task (target with no feedback), both the left and right hemispheres are in the same cluster; therefore, the values of *k* are the same. The significantly activated regions in the motor cortex (left and right hemispheres) are also within the same cluster during the TT task.

### 4.4 Dynamic causal model

#### 4.4.1 Network connection

**Figure 7:**
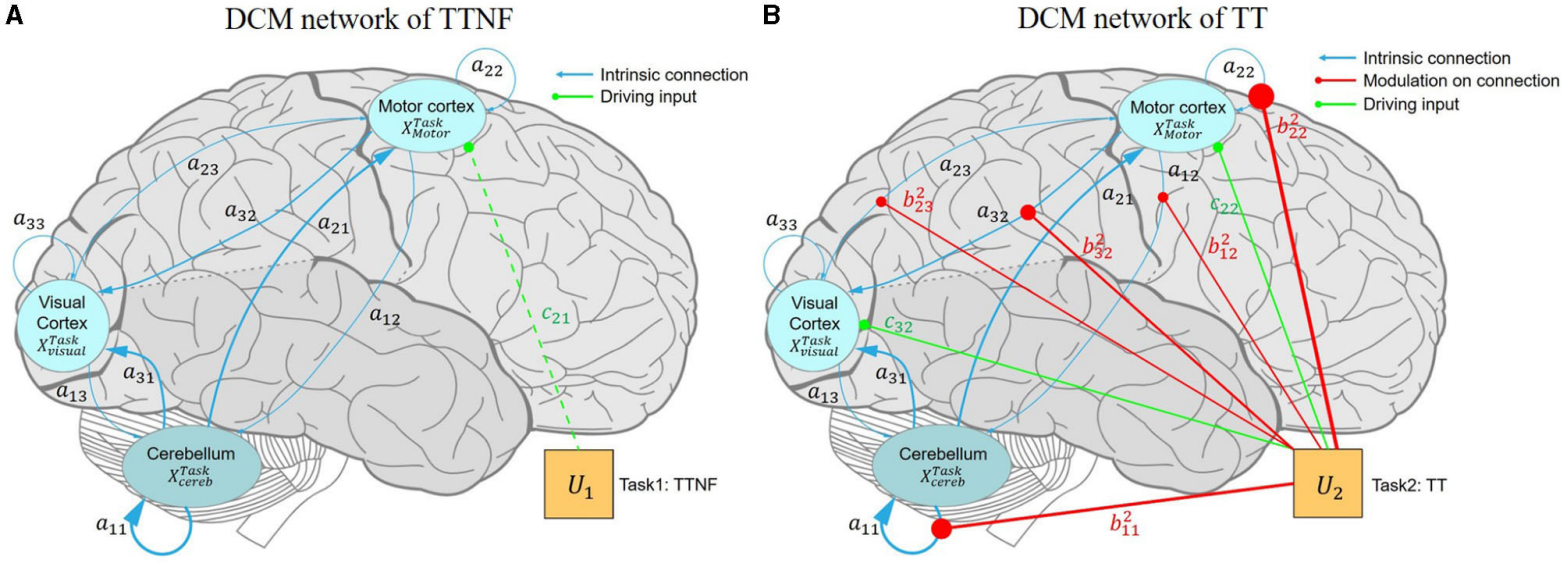
Effective connectivity of the motor control model among the motor cortex, visual cortex, and cerebellum. The blue, red, and green lines represent the intrinsic connections, modulatory connections, and driving inputs, respectively. **(A)** DCM model driven by the TTNF task. The intrinsic connectivity shows strong inhibition in CBR → CBR (*a*_11_ = –0.885 Hz) and ML → VL (*a*_32_ = 0.509 Hz) as bolder blue lines, which indicate strong self-inhibition in the activation of CR, and suppression to the activation of VL from ML. Weaker connectivity is shown by thinner lines as for ML → CBR (*a*_12_ = –0.070 Hz), suggesting that ML does not dominate motor control during simple motor behavior like the TTNF task. The finding that the driving input of TTNF only slightly affects ML *c*_21_ = 0.083 Hz also supports this result, indicating that ML was not sensitive to the stimuli (movement of the target sphere). TTNF was set as the basic condition and all the parameters in matrix **B**^1^ were set to 0, so no modulatory connection (red lines) are shown for this task. **(B)** DCM model driven by the TT task. The modulatory connection showed strong enhancements to the connections of CBR → CBR ($b_{11}^{2}$ = 1.180 Hz) and ML → ML ($b_{22}^{2}$ = 1.798 Hz), indicating stronger self-inhibition in CBR and ML for adaptive visuomotor control. In addition, the negative modulation of ML → VL ($b_{32}^{2}$ = –0.606 Hz) decreased the suppression from ML to VL (*a*_32_ = –0.509 Hz), indicating significantly activated clusters in the visual cortex. The negative modulation of ML → CBR ($b_{12}^{2}$ = –0.451 Hz) compared with that of the intrinsic network (*a*_12_ = –0.070 Hz) also showed that ML decreased the suppression of CBR and led to increased activity in CBR. These inhibitions from the motor cortex to the visual cortex and cerebellum represent corrections of motor commands from the high-level controller. The inhibition from the visual cortex to the motor cortex shows the effects of visual feedback to motor predictions. The driving input of TT affects both the motor cortex (*c*_22_ = 0.409 Hz) and the visual cortex (*c*_32_ = 0.379 Hz). CBR, right cerebellum; ML, left motor cortex; VL, left visual cortex; DCM, dynamic causal model; TT, target with feedback task; TTNF, target with no feedback.

As described in the methods section, we first built a fully connected model and then simply pruned away any parameters from the PEB that did not contribute to the model evidence. We averaged the estimated connection strengths at the group level. The final model obtained after Bayesian model reduction is shown in Figure 7. The average explained variance of participant-level DCMs was 35.75%±14.52%, ranging from 7.84% to 53.49%. The estimated parameters of matrices **A**, **B**, and **C** are listed in Table 4.

**Table 4:** Connectivity strength (posterior probability) during TTNF and TT obtained by Bayesian model averaging of PEB model parameters.

**Connection type**	**Mean**	**Improved RMSE**	**Mean MDF**
**Endogenous parameters: Matrix A (Hz)**			
*a*_21_: CBR → ML	0.697 (1.00)	-	-
*a*_31_: CBR → VL	0.732 (1.00)	-	-
*a*_12_: ML → CBR	–0.070 (1.00)	-	-
*a*_32_: ML → VL	–0.509 (1.00)	-	-
*a*_13_: VL → CBR	0.277 (1.00)	-	-
*a*_23_: VL → ML	0.217 (1.00)	-	-
**Self-inhibition parameters: Matrix A (Hz)**			
*a*_11_: CBR → CBR	–0.885 (1.00)	-	-
*a*_22_: ML → ML	0.267 (1.00)	-	-
*a*_33_: VL → VL	–0.148 (0.93)	-	-
**Modulatory parameters: Matrix B (Hz)**			
$b_{11}^{2}$: CBR → CBR	1.180 (1.00)	-	0.960 (1)
$b_{21}^{2}$: CBR → ML	-	-	-
$b_{31}^{2}$: CBR → VL	-	-	-
$b_{12}^{2}$: ML → CBR	-0.451 (1.00)	-	-
$b_{22}^{2}$: ML → ML	1.798 (1.00)	0.577 (0.98)	-
$b_{32}^{2}$: ML → VL	–0.606 (1.00)	-	-
$b_{13}^{2}$: VL → CBR	-	-	-
$b_{23}^{2}$: VL → ML	–0.399 (0.95)	0.189 (0.71)	-
$b_{33}^{2}$: VL → VL	-	-	-
**Input parameters: Matrix C (Hz)**			
*c*_11_: TTNF → CBR	-	-	-
*c*_21_: TTNF → ML	0.083 (0.55)	-	-
*c*_31_: TTNF → VL	-	-	-
*c*_12_: TT → CBR	-	-	-
*c*_22_: TT → ML	0.409 (1.00)	-	-
*c*_32_: TT → VL	0.379 (1.00)	-	-

Between-region connections are in units of Hz. Self-inhibition parameters are the log of scaling parameters that multiply up or down –0.5 Hz (the default self-connection value). Posterior probabilities are given in brackets. This table includes all parameters of the 256 best models during Bayesian model averaging. *n* = 13. In this study, we set TTNF as the basic condition. Therefore, all the parameters in matrix **B**^1^ were set as 0 and are not shown in this table. CBR, right cerebellum; ML, left motor cortex; VL, left visual cortex; PEB, parametric empirical Bayes; TT, target with feedback task; TTNF, target with no feedback; improved RMSE, decreased right root mean square error (TTNF vs. TT) in the behavior analysis.

The intrinsic network (matrix **A**) showed positive connections from CBR to ML (*a*_21_ = 0.697 Hz, PP = 1) and to VL (*a*_31_ = 0.732 Hz, PP = 1) and positive connections from VL to CBR (*a*_13_ = 0.277 Hz, PP = 1) and to ML (*a*_23_ = 0.217 Hz, PP = 1). These findings indicate that CBR and VL increased the activity in the other regions. Meanwhile, we found that the connections from ML to CBR (*a*_12_ = –0.070 Hz, PP = 1) and to VL(*a*_31_ = –0.509 Hz, PP = 1) were negative, showing that inhibition from ML decreased activity in CBR and VL. The input parameter results (matrix **C**) show the sensitivity of the selected regions to the two driving inputs, namely, tasks TTNF and TT. The results show that TTNF probably affected the motor cortex with a lower sensitivity of *c*_21_ = 0.083 Hz (PP = 0.55), while task TT affected both the motor cortex (*c*_22_ = 0.409 Hz, PP = 1.00) and the visual cortex (*c*_32_ = 0.379 Hz, PP = 1.00).

The network connectivity was modulated strongly during the TT task (matrix **B**). We found that ML → CBR ($b_{12}^{2}$ = –0.451 Hz, PP = 1) and ML → VL ($b_{32}^{2}$ = –0.606 Hz, PP = 1) were negatively modulated, which decreased the inhibition from ML to CBR and VL in the intrinsic network. These modulations could lead to increasing activity from ML to CBR and VL, showing that ML dominated the motor control as a higher-level controller. This can also explain why significantly activated clusters were found in only the visual cortex during TT. We also found negative modulation from VL to ML ($b_{23}^{2}$ = –0.399 Hz, PP = 0.95) indicating less activation from VL to ML. However, no modulation was found from CBR to ML or VL.

We assessed the effects of individual differences by including improvement in tracking performance (RMSE) and muscle activation (mean MDF) as covariates in our model. We identified two connections, ML → ML (0.577 Hz, PP = 0.98) and VL → ML (0.189 Hz, PP = 0.71), that were positively related to individual differences in improved tracking performance (RMSE). These results show that the improved tracking performance was positively related to the enhanced connection from VL to ML. Meanwhile, CBR → CBR connection (0.960 Hz, PP = 1.00) was found to be positively related to mean MDF.

#### 4.4.2 Cross-validation of predictions

We used LOOCV to examine whether the network connections derived from group analysis could predict individual differences. In particular, we tested the improved RMSE in behavior analysis and mean MDF, both after normalization (mean-centered and z-scored). The tests of improved RMSE were made based on the ML → ML and VL → ML connections because the two connections were found to be positively correlated with the improved RMSE, as explained in the previous subsection. The test of mean MDF was predicted using modulation of ML → CBR by visual feedback (task TT). This is because we were interested in whether we could predict the change in muscle activity from the modulation of the cerebellum by the motor cortex.

**Figure 8:**
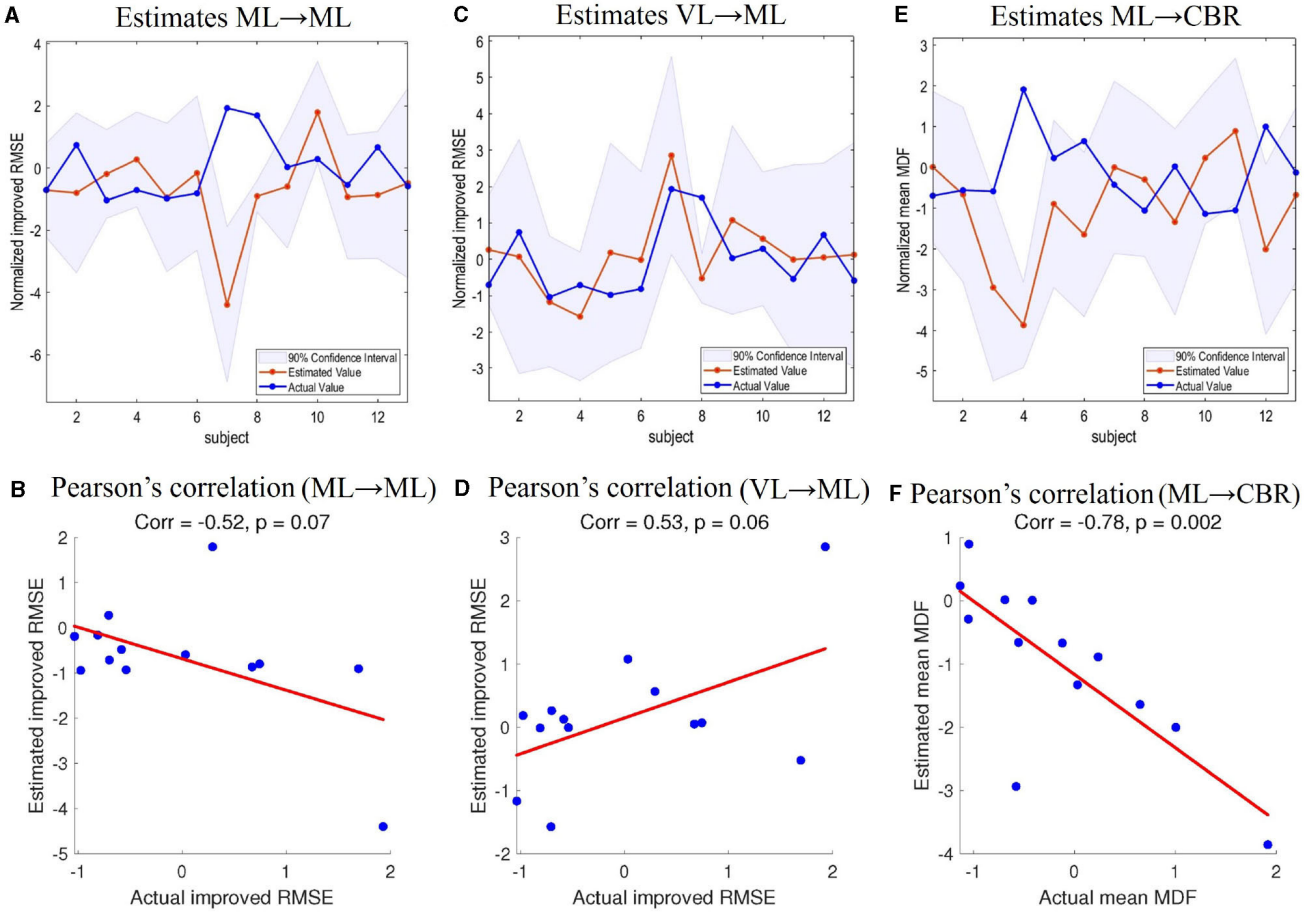
Leave-one-out cross-validation. **(A)** Estimations of improved RMSE based on ML → ML connectivity. **(B)** Pearson's correlation coefficient between improved RMSE estimations based on ML → CBR connectivity and actual values. **(C)** Estimations of improved RMSE based on VL → ML connectivity. **(D)** Pearson's correlation coefficient between improved RMSE estimations based on VL → ML connectivity and actual values. **(E)** Estimations of muscle MDF based on ML → CBR connectivity. **(F)** Pearson's correlation coefficient between estimated muscle MDF based on ML → CBR and actual muscle MDF. CBR, right cerebellum; ML, left motor cortex; VL, left visual cortex; RMSE, root mean square error; MDF, median muscle frequency.

In Figures 8A, C, E, the lines with red circles show the predicted values of improved RMSE and mean MDF for each left-out subject. The shaded areas are the 90% credible interval of the prediction and the lines with blue circles represent the actual values. Figures 8B, D, F shows the scatterplots of the actual and predicted values along with the Pearson's correlation coefficients. In the LOOCV of improved RMSE based on ML → ML and VL → ML, 11/13 and 12/13 participants had their actual value of improved RMSE within the estimated 90% credible interval. Pearson's correlation coefficients were –0.52 (*p* = 0.07) and 0.53 (*p* = 0.06) respectively. The LOOCV of mean MDF also showed that 10/13 participants had their actual value within the 90% credible interval. Pearson's correlation coefficient was –0.78 (*p* = 0.002). Therefore, we can conclude that the DCM can predict the improved tracking performance (RMSE) and the muscle activity characteristics (mean MDF), although there is still variability.

## 5 Discussion

Rather than a functional understanding of individual brain regions in isolation, our results suggest that the hierarchical model could functionally explain human motor control that effectively integrates incoming visual input and minimizes tracking errors. We designed a closed-loop visuomotor control task with moving a target in contrast to previous visuomotor tasks (13). This design enabled us to study the motor-cerebellar interaction with integrated visual feedback during target tracking motor control. Our application of recent methodological advancements in the field of human motor control enables a more thorough characterization of the underlying neural mechanisms. These insights can potentially inform the development of novel motor control schemes by translating research findings into practical applications.

### 5.1 Behavior analysis and DCM evaluation

At the behavioral level, our study demonstrated a significant reduction in the RMSE of tracking performance in the TT task compared with the TTNF task. This finding indicates that participants made a conscious effort to minimize the error in their tracking in the TT task as instructed, which is consistent with prior research suggesting that trajectory deviations are corrected only when they interfere with task performance (1). The visual feedback in TT allowed participants to adjust their motor output and minimize the difference between the target and feedback spheres.

We used the behavioral results in the DCM to correct for participants' individual differences. We found a stronger connectivity strength of ML → ML ($b_{22}^{2}$ = 0.577 Hz; probability: 0.98; covariates: normalized improved RMSE). We used the DCM and made predictions about the improved RMSE based on the ML → ML connection. These results suggest that increased activation in the motor cortex was related to improved RMSE. Stronger activation in the motor cortex might lead to better tracking performance. We also found that the enhanced connectivity strength of VL → ML ($b_{23}^{2}$ = 0.189 Hz; probability: 0.71; covariates: normalized improved RMSE) could predict the improvement of tracking performance. Based on the findings in modulated ML → ML and VL → ML connections, we further verified that the two DCM connections could estimate the improved RMSE (TT vs. TTNF) in the behavior analysis. The LOOCV of ML → ML and VL → ML connections showed that the modulated connectivity strength of our DCM could predict individual differences in tracking performance. To summarize, the modulation of connection strength suggests that visual feedback can help minimize the differences between predictions and actual motor outcomes by enhancing the connectivity strength from VL to ML. Our study sheds light on how functional brain network modulation affects motor control and provides a quantitative method for evaluating the effects of modulation on tracking performance.

### 5.2 DCM connectivity

Based on the recognition that human sensorimotor control can be explained using a hierarchical model (26, 27), we built a DCM that included two levels of neural feedback control with the motor cortex regions as a high-level controller that monitors progress and improves performance and the cerebellum as a low-level controller that provides automatisms (2). Compared with previous analysis of the cerebellar-premotor cortex interactions (13), we also considered the interactions with the visual cortex underlying visuomotor control. Our aim was to uncover functional aspects of the motor-visual-cerebellar circuitry that could explain fast-correction processing in target tracking.

The positive and negative values of connectivity strength parameters between brain regions respectively indicated enhancement or suppression of the connection. Our analysis revealed a negative value of ML → VL (*a*_32_ = –0.509 Hz) in the intrinsic network, indicating suppression from the motor cortex to the visual cortex. This finding suggests that participants tended to ignore visual stimulation from the target and suppress the visual cortex when no visual feedback was provided. This result was consistent with earlier findings from studies in monkeys (28), which found that the primary visual cortex of monkeys was selectively suppressed when high saliency stimuli were not seen. Our result was also supported by the fMRI group analysis results showing no activation in the visual cortex during the TTNF task (Figure 6). Another possible explanation was that participants might rely more on proprioception information to follow the target's movement, as proprioception has been found to be primarily used for online corrections during rapid, unseen movements toward visual targets (29). Our results were also consistent with the study by Bagesterio et al., which found that vision was mainly used for planning movement distance while proprioception dominated online corrections during rapid, unseen movements toward visual targets. The modulatory parameters show how the motor control changed according to visual feedback. During TT, the ML → VL connectivity ($b_{32}^{2}$ = –0.606 Hz) was more strongly suppressed than in the intrinsic network during TTNF (*a*_32_ = –0.509 Hz), which could be interpreted as negative modulation from the motor cortex activity. The observed suppression from the motor cortex to the visual cortex would be consistent with the hierarchical message passing model (9).

The connection from CBR to ML was positive during TTNF, similar to the results of Pool et al. (30), who found that interactions between the cerebellum and premotor areas are positive during simple motor behavior. Our finding suggests that the cerebellum contributed to fast automated motor control during TTNF as the lower-level controller, consistent with the functional role of the cerebellum in the cerebro-cerebellar loop as reviewed by Tanaka et al. (7). The negative modulation of the ML → CBR connection ($b_{12}^{2}$ = –0.451 Hz > *a*_12_ = –0.070 Hz) shows that ML decreased suppression of CBR and therefore increased the activity in CBR. It also supports the interpretation that the motor cortex acted as a higher-level controller when motor adjustment was necessary during TT. The validation of the modulated ML → CBR connection also demonstrated that it could be used to estimate the effects on muscle activity (see LOOCV of mean MDF in Figure 8F). The individual performance in terms of muscle activity also showed a correlation with positive modulation of cerebellar activity. The enhancement of CBR → CBR (0.980 Hz) represented that higher activation in the cerebellum was related to higher mean MDF in participants. Our results provide valuable insights into the functional architecture of the motor-visual-cerebellar circuitry and shed light on the mechanisms underlying fast-correction processing in target tracking. They also illustrate the utility of DCM in quantifying the effective connectivity changes caused by visuomotor tasks, which could be useful for developing more efficient rehabilitation tasks for enhancing the motor-visual and motor-cerebellar connections.

### 5.3 Study limitation

In this study, we measured the MRI and EMG using the same experimental protocol separately because the EMG sensors could not be used in the MRI room. However, it would be hard to quantitatively evaluate whether the participants performed similar movements in the two measurements. Raz et al. reported that vision and posture play a major role in influencing behavior and MRI results, and a mock MRI scanner could help address the limitations of separate measurements (31). Therefore, we developed a dummy MRI scanner, which aimed to recreate the MRI environment, especially in terms of posture and vision when measuring the EMG. We fixed the body posture and positions of the head, elbows, and wrists to restrict the movement. While this may not completely replicate muscle activities, it is designed to generate very similar results under identical behavioral restrictions. Similar upper limb motion measurements in MRI and EMG were published in a spinal cord MRI study (32).

There are several ways in which our work could be extended. For example, additional shorter-duration measurements (i.e., an event-based experimental design) could be considered for capturing distance-sensitive areas within the parietal and frontal cortex regions, which may warrant inclusion in the hierarchical model. It would also be worthwhile to improve the design of the TTNF task by adding a static “feedback” circle to keep the number of visual stimuli the same in both the TT and TTNF tasks and prevent unexpected activation in the visual cortex.

## 6 Conclusion

In this study, we investigated the mechanisms of hierarchical motor control in humans by combining experimental behavior analysis and muscle activity with brain imaging techniques. We first used behavior analysis to evaluate the tracking performance during different voluntary hand motions. Our exploration commenced with a meticulous examination of behavioral data, allowing us to scrutinize tracking performance across various voluntary hand motions. This analysis uncovered notable disparities in motor control strategies, highlighting the multifaceted nature of this cognitive processing.

Subsequently, we explored the realm of neural dynamics, utilizing DCM and PEB to quantitatively assess neural activity interactions within the motor, visual, and cerebellar cortices. Our findings illuminated substantial differences in behavioral control performance between tasks executed with and without visual feedback. Notably, the presence of visual feedback resulted in a heightened modulated suppression pathway from the motor cortex to both the cerebellum and the visual cortex, emphasizing the pivotal role of visual input in shaping motor control.

Furthermore, our study delved into the individual nuances of motor control by scrutinizing the modulation of connection strength within the VL → ML, ML → ML, and ML → CBR pathways under the influence of visual feedback. This analysis provided insights into the neural signatures responsible for individual variations in tracking performance and muscle activity. The results of this research offer fresh perspectives on the mechanisms underpinning hierarchical motor control, particularly in the context of visual feedback integration. By deciphering the intricacies of human motor control, our work is expected to contribute to the advancement of personalized interventions and technologies, ultimately enhancing human capabilities and quality of life.

## Data availability statement

The raw data supporting the conclusions of this article will be made available by the authors, without undue reservation.

## Ethics statement

This study was conducted in accordance with the Declaration of Helsinki and approved by the Ethical Review Board of the RIKEN Research Institute (code of the ethical approval: RIKEN-W3-2021-023, date of approval: October 14th, 2021). The studies were conducted in accordance with the local legislation and institutional requirements. The participants provided their written informed consent to participate in this study. Written informed consent was obtained from the individual(s) for the publication of any potentially identifiable images or data included in this article.

## Author contributions

NY: Conceptualization, Investigation, Methodology, Software, Validation, Writing – original draft, Writing – review & editing, Data curation, Formal analysis. SU: Data curation, Formal analysis, Methodology, Writing – review & editing. ÁC-G: Conceptualization, Data curation, Methodology, Writing – review & editing. SO: Data curation, Methodology, Writing – review & editing. HT: Methodology, Writing – review & editing, Conceptualization. JL: Conceptualization, Funding acquisition, Supervision, Writing – review & editing. SS: Conceptualization, Funding acquisition, Supervision, Writing – review & editing, Project administration.
